# Probing the action of a novel anti-leukaemic drug therapy at the single cell level using modern vibrational spectroscopy techniques

**DOI:** 10.1038/s41598-017-02069-5

**Published:** 2017-06-01

**Authors:** Joanna L. Denbigh, David Perez-Guaita, Robbin R. Vernooij, Mark J. Tobin, Keith R. Bambery, Yun Xu, Andrew D. Southam, Farhat L. Khanim, Mark T. Drayson, Nicholas P. Lockyer, Royston Goodacre, Bayden R. Wood

**Affiliations:** 1 Manchester Institute of Biotechnology and School of Chemistry, University of Manchester, Manchester, M1 7DN United Kingdom; 2 Biomedical Research Centre, School of Environment and Life Sciences, University of Salford, Salford, M5 4WT United Kingdom; 3 Centre for Biospectroscopy and School of Chemistry, Monash University, Clayton, Victoria 3800 Australia; 4 Australian Synchrotron, 800 Blackburn Road, Clayton, Victoria 3168 Australia; 5 School of Biosciences, University of Birmingham, Birmingham, B15 2TT United Kingdom; 6 Institute of Immunology and Immunotherapy, University of Birmingham, Birmingham, B15 2TT United Kingdom

## Abstract

Acute myeloid leukaemia (AML) is a life threatening cancer for which there is an urgent clinical need for novel therapeutic approaches. A redeployed drug combination of bezafibrate and medroxyprogesterone acetate (BaP) has shown anti-leukaemic activity *in vitro* and *in vivo*. Elucidation of the BaP mechanism of action is required in order to understand how to maximise the clinical benefit. Attenuated total reflectance Fourier transform infrared (ATR-FTIR) spectroscopy, Synchrotron radiation FTIR (S-FTIR) and Raman microspectroscopy are powerful complementary techniques which were employed to probe the biochemical composition of two AML cell lines in the presence and absence of BaP. Analysis was performed on single living cells along with dehydrated and fixed cells to provide a large and detailed data set. A consideration of the main spectral differences in conjunction with multivariate statistical analysis reveals a significant change to the cellular lipid composition with drug treatment; furthermore, this response is not caused by cell apoptosis. No change to the DNA of either cell line was observed suggesting this combination therapy primarily targets lipid biosynthesis or effects bioactive lipids that activate specific signalling pathways.

## Introduction

Acute myeloid leukaemia (AML) is an aggressive cancer that leads to a build-up of non-differentiated, dysfunctional cells from the myeloid lineage. Without treatment, patients can die from impaired haemopoiesis within weeks of diagnosis and unfortunately responses to current treatments and overall survival of patients post-treatment generally remains poor^1^.

Current AML treatment is centred on short cycles of intensive cytotoxic chemotherapy to kill the cancer cells, however, this treatment can disrupt the production of healthy blood cells. Continuous use of intensive chemotherapy is precluded because of the high levels of toxicity that affect haemopoiesis and cell division, which is important in maintaining the integrity of the body’s mucosal surfaces. This dose-limiting toxicity prevents escalation of chemotherapy to treat resistant disease and precludes its use in many older frailer individuals who account for a high proportion of AML patients^2^. Accordingly, there is an urgent need for low toxicity anti-AML therapies that can be used both as an adjunct to chemotherapy and in between chemotherapy cycles. Continuous low toxicity therapy is a novel strategy to manage AML by providing good quality survival even without total eradication of the disease.

Drug redeployment (repurposing) represents an attractive therapeutic option that involves the use of existing drugs for treatments which they were not originally intended and has the potential to offer less toxic therapies than chemotherapy^3^. The redeployed combination of the cholesterol lowering drug, bezafibrate, and the female contraceptive, medroxyprogesterone acetate (combination denoted BaP) has anti-leukaemic and anti-lymphoma activity without haematological toxicity both *in vitro* and in patients^4–7^. Mass spectrometry lipidomics of BaP treated AML cell lines has recently demonstrated that BaP slows *de novo* fatty acid biosynthesis, particularly the synthesis of monounsaturated fatty acids, which plays a key role in BaP-related AML cell killing^8^. This has relevance in the wider cancer field where tumour cells often have up-regulated fatty acid synthesis and inhibition of this process leads to tumour cell death^9, 10^. The effect of BaP on *de novo* lipogenesis also led to a broad change in the overall phospholipid acyl chain composition, which may play a role in cell killing^8^. A spectroscopic investigation of the action of BaP at the single cell level offers an original insight into the mode of action of this combination therapy in which living cells can be monitored with BaP treatment. This eliminates the need for the extraction of lipids or metabolites from cells; thereby giving a more holistic picture of the cell biochemistry. Employing high-resolution techniques such as Fourier Transform infrared (FTIR) spectroscopy and Raman microspectroscopy provides new insights into the drug mechanism of action at sub cellular resolution.

FTIR and Raman microspectroscopy have both developed significantly in the last decade as powerful tools for probing the molecular structure of biological specimens such as tissue, cells and serum^11–19^. They are complementary techniques with different selection rules - FTIR spectra arise from the absorption of radiation from functional groups with a permanent dipole moment; whereas Raman spectra result from the inelastic scattering of light from molecules where the dipole moment is induced by the incident laser which causes a change in the intrinsic polarizability of the molecule. Molecules or functional groups that strongly scatter Raman light tend be more symmetric and chromophoric, whereas strong IR absorbers are usually more asymmetric in terms of their electronegativity when vibrating. The techniques are capable of providing a rapid, rich biochemical ‘fingerprint’, which on interpretation are extremely informative in both a research and more recently, a diagnostic setting^20–24^. Probing drug-cell interactions with spectroscopic techniques has become increasingly popular and can contribute to the understanding of the mode of action of the drug at a cellular level^25, 26^. The majority of spectroscopic cellular studies reported to date are on cells that have been chemically fixed and are therefore often in a dehydrated state^17, 18, 23, 27–29^. Fixation aims to preserve the structural and biochemical constituents of cells in as close to *in vivo* conditions as possible and is widely accepted in the field of spectroscopy^29^. It also has the advantage of preventing cells from moving outside of the field of view or away from the irradiation source during cell imaging. However, cell dehydration during fixation changes the conformation of the DNA from the B-form to the more disordered A-form^30^ which renders the A-form DNA bands weaker and broad when compared to B-form bands making them difficult to discern from other macromolecules like protein, RNA and carbohydrates^30, 31^. This is pertinent when trying to assess whether or not there has been intercalation of a drug treatment with the DNA resulting in either conformational change or denaturing of the molecule and exemplifies that there are advantages gained through the study of live cells over that of fixed cells. Further disadvantages associated with fixation are the use of chemicals which can interfere with the inherent biochemical signature of the cell, thus potentially altering the spectrum^32^ and that light scattering effects (due to the difference in refraction indices between the cell and surroundings) are commonly observed in FTIR, which must be subsequently corrected for during spectral interpretation. Thus, there are significant benefits to be had for probing a cell in its hydrated state for a more accurate view of the nature of intracellular biochemical species in a tested physiological condition. Employing the high fluency and superior brightness of a synchrotron beam^33^ enables such *in vivo* analysis to be performed in real time with cells remaining in their growth medium, thereby eliminating the requirement for any potentially detrimental sample preparation. The ability to probe single cells, one by one is extremely desirable, ensuring information obtained is specific to the living cell in question, rather than that averaged over a heterogeneous cell population, which can also include cell debris and cells that are undergoing cell death.

To date, biochemical and morphological classification of healthy *versus* diseased cell lines has been well demonstrated with FTIR and Raman spectroscopies^34, 35^ and in the field of leukaemia, spectroscopic studies have investigated leukaemia cell classification^27, 36, 37^ drug cytotoxicity^38^ and leukemic cell apoptosis^39, 40^. Only a few studies have employed spectroscopic techniques to specifically probe the nature of AML^41–43^ and as far as we are aware, there are no reports which combine FTIR and Raman spectroscopy to study targeted anti-AML drug-cell interactions. Furthermore, typical cells reportedly studied in many drug-cell interaction situations are adherent cells, which are amenable to growth onto a substrate for analysis and are therefore somewhat easier to manipulate than suspension cells for the purpose of single cell microspectroscopic analysis. Here we present for the first time, a spectroscopic interpretation of the action of the recently reported BaP drug therapy in two AML suspension cell lines, HL60 and K562. Cells reported here were analysed live (synchrotron-FTIR), dehydrated (ATR-FTIR) and fixed (Raman); a diversity which contributes to a broad spectroscopic picture of drug action. The large number (>800) of single cells reported here gives further confidence to the power of the techniques, as described by statistical models for data interpretation. These data give an *in situ* interpretation of the effect of BaP treatment on AML cells.

## Results and Discussion

Direct infusion mass spectrometry analysis of drug-treated AML cells shows that bezafibrate and medroxyprogesterone acetate are taken up by the cells as illustrated in Supplementary Figure S1. The combination of these drugs (denoted BaP) has a potent anticancer effect on the HL60 and K562 cell lines that are used in this study^7, 8^.

### Overview of FTIR Spectra

**Figure 1:**
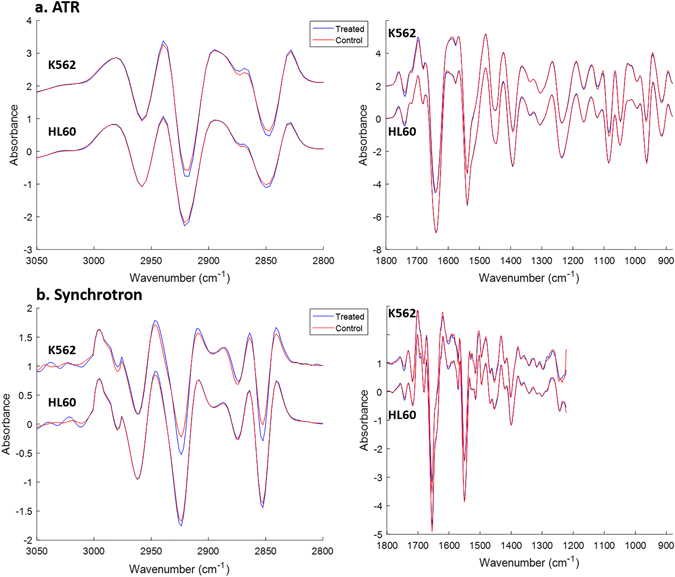
Normalised, mean second derivative spectra from 9 replicates for ATR analysis (**a**) of HL60 and K562 control and BaP treated cells and 3 replicates for each of 2 trials for S-FTIR analysis (**b**), incorporating a total of 441 individual cells (HL60) and 385 individual cells (K562). The high wavenumber (3050–2800 cm^−1^) region is predominated by the *ν*_*as*_(CH_3_), *ν*_*as*_(CH_2_), *ν*_*s*_(CH_3_) and *ν*_*s*_(CH_2_) stretches at 2955, 2920, 2870 and 2850 cm^−1^ respectively, which arise from cellular lipid species. Synchrotron data were not collected below *ca* 1200 cm^−1^ and the plot is generated so that the x-axis is on the same wavenumber scale.

Data are presented for both ATR and S-FTIR analyses. Cells analysed by ATR-FTIR were sampled from a heterogeneous cell pellet which predominately consisted of viable cells, but was also likely to contain cells in varying stages of necrosis and cell debris from cells which may have undergone apoptosis in culture. A significant advantage of this technique is that it is easily accessible, portable and inexpensive. It also minimises scattering and other baseline contributions, as shown in the raw spectra presented in Supplementary Figure S2. However, cells were dehydrated, thus limiting the biochemical information that can be derived from resulting spectra. Exploiting the superior properties of the synchrotron beam^33^ allows transmission of light through aqueous environments and the two windows of the sample holder, thus enabling the analysis of live single hydrated cells. Cells were clearly visible under the microscope to confirm their viability and a randomised selection of single cells was selected from each aliquot of cells in this study. Averaged second derivative spectra for K562 and HL60 cells analysed with both techniques are shown in Fig. 1 (second derivative spectra shown to enhance spectral features). The C-H stretching region (3050–2800 cm^−1^) as well as the lower wavenumber cell fingerprint region (shown as 1800–900 cm^−1^ for ATR-FTIR and 1800–1200 for S-FTIR) are overlaid for control and 24 hour BaP treated cell spectra for both ATR-FTIR (Fig. 1a) and synchrotron-FTIR (Fig. 1b) analysis of each cell line, respectively.

Spectra measured by ATR were acquired for air-dried cell pellets, whereas the spectra obtained by S-FTIR were only of live hydrated cells. The difference in hydration states significantly affects both band intensity and width. In the hydrated state the DNA is in the B-DNA form and the bands appear sharper and more intense compared to when the cells are dehydrated and the A-DNA form dominates, which is more disordered compared to B-form^31^. The difference in hydrogen bonding between hydrated and dehydrated cells also affects the width and position of the amide modes from proteins.

### Orthogonal partial least squares-discriminant analysis of FTIR data

**Figure 2:**
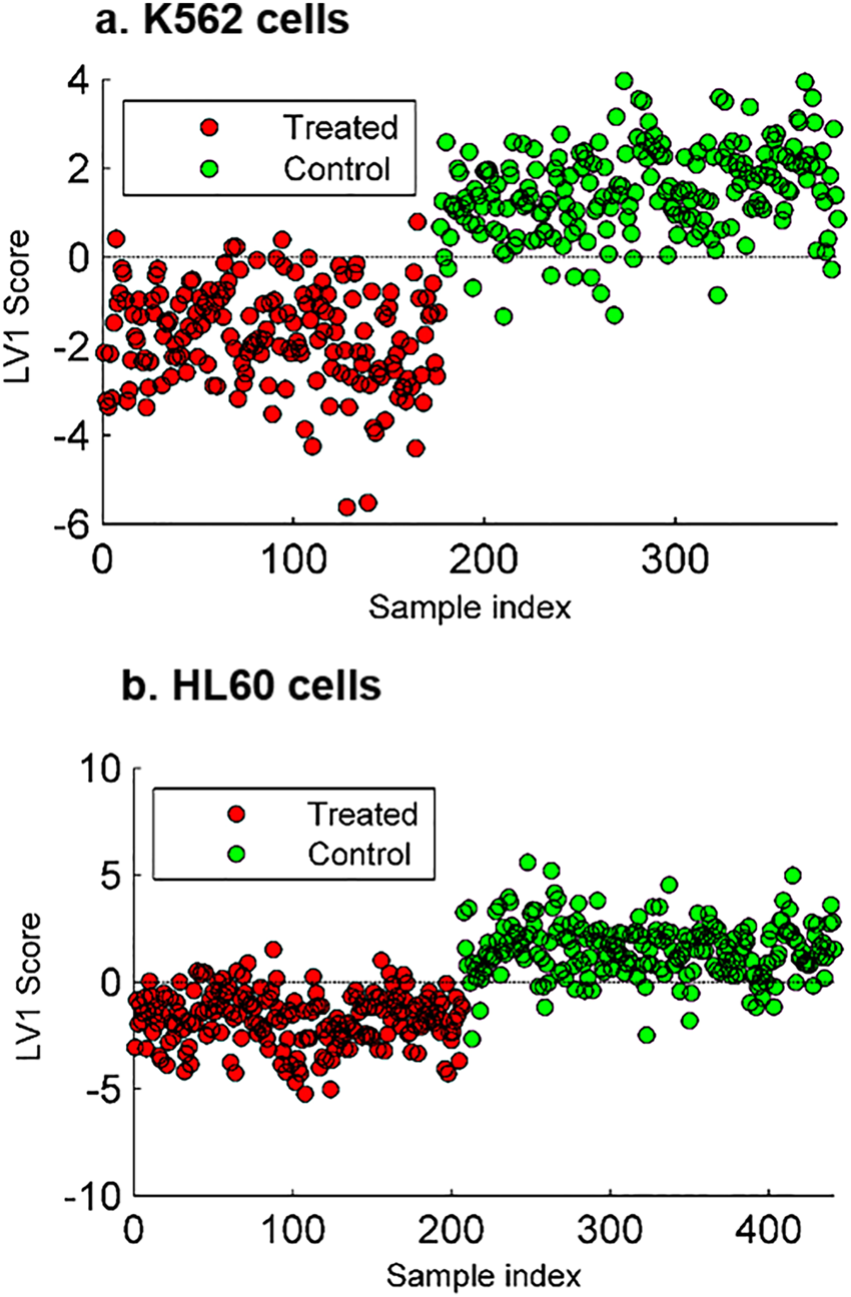
Scores plots for the first oPLS-DA component for S-FTIR spectra of control (green) and BaP treated (red) K562 (**a**) and HL60 (**b**) cells. Each point represents a K562 single cell (**a**) and HL60 single cell (**b**).

Orthogonal partial least squares-discriminant analysis (oPLS-DA)^44^ was performed using the 3059–2715 cm^−1^ and the 1819–1223 cm^−1^ regions for the synchrotron data and using the 3133–2806 cm^−1^ and the 1840–862 cm^−1^ regions for the ATR data for control and 24 hour drug treated cell spectra. Figure 2 shows the scores of the first oPLS-DA latent variable (LV) for each cell type, K562 (Fig. 2a) and HL60 (Fig. 2b) with LV1 clearly distinguishing drug treatment (red) from control cells (green). The same trend was observed for ATR spectra and is shown Supplementary Figure S3. Although the LV1 calibration values are not good indicators of the classification performance due to overfitting issues, they are necessary to interpret the direction of the oPLS-DA first loading vector. A more reliable estimation of the model quality was obtained by the cross validation results (Venetian blinds, 10 splits), which indicated a good classification performance in the four cases, given error rates of 15% (4LVs), 0% (3LVs), 15% (2LVs) and 11% (1LV).

**Figure 3:**
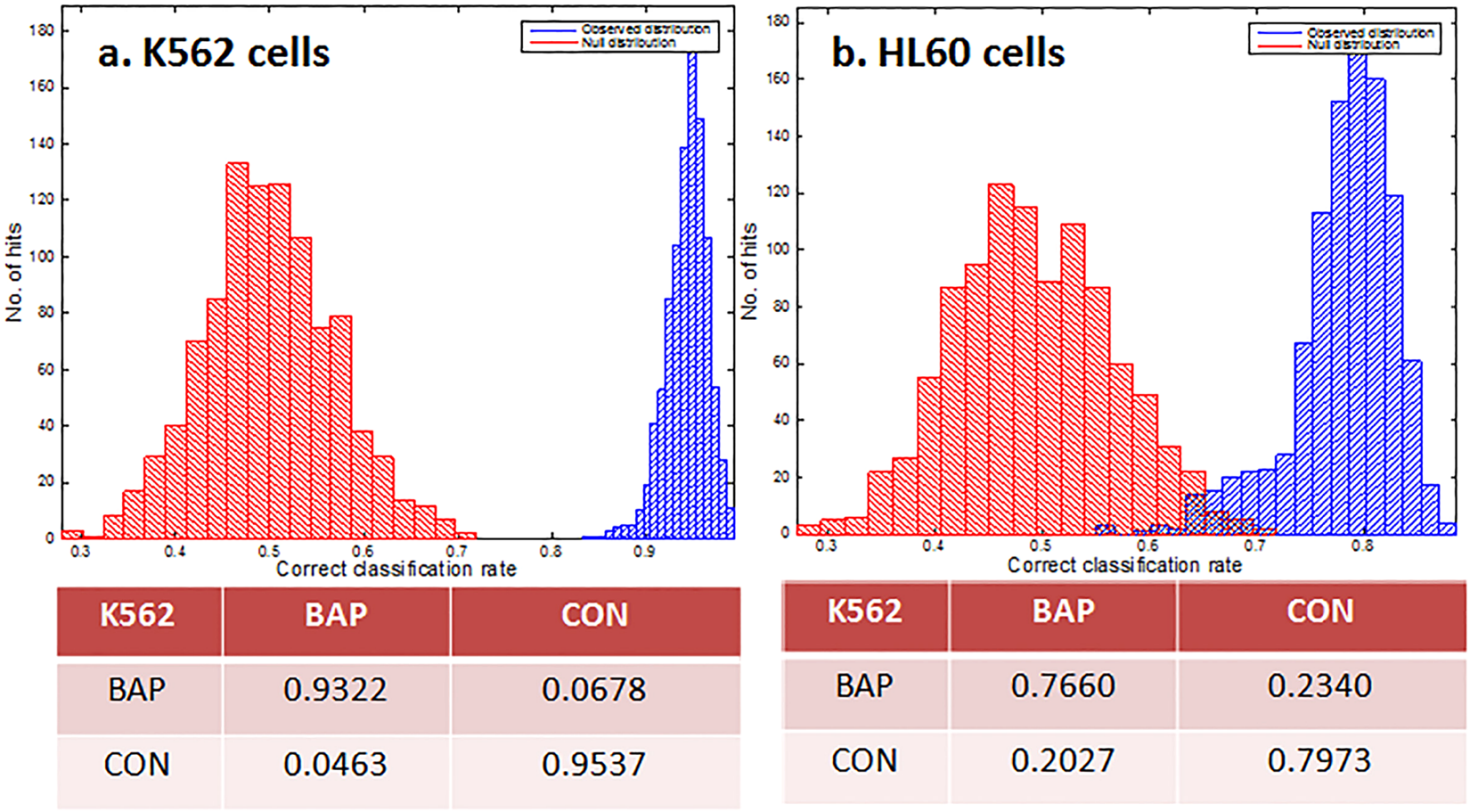
Classification model for PLS-DA of S-FTIR analysis of whole cell populations for K562 (**a**) and HL60 (**b**) cells showing classification rates for real (blue) and random (red), with a CCR of 1 being a 100% correct classification. The embedded tables are the average confusion matrices with rows representing predicted classification and columns representing experimental.

Further validation of the oPLS-DA model was obtained by using partial least squares discriminant analysis (PLS-DA). The classification model for the PLS-DA computed across the spectral region 3100–1100 cm^−1^ for all S-FTIR cells analysed, with no outliers removed, can be seen in Fig. 3. Bootstrapping was performed 1000 times to permute whether the cell was drug treated or not. The output shows two discrete distributions; a ‘real’ (in blue) and ‘null/random’ (in red) model, these models have good correct classification rates (CCR) of an average of 94.36% for K562 and 78.14% for HL60 cells.

When comparing data acquired between the two cell lines, it is apparent that HL60 cells (which are on average typically smaller than K562 cells) show a greater distribution in spectra than K562 cells. This is understandable, given that the aperture size employed and average size of HL60 cells is comparable, thereby a portion of HL60 cells at less than 10 µm in diameter would contribute non –cellular features to the spectra and are were more likely to appear as outliers.

### FTIR spectral interpretation

A visual inspection of the ATR spectra (Fig. 1a) reveals an apparent change in the intensity of signals in the high wavenumber (3050–2800 cm^−1^) region, predominated by the *ν*_*as*_(CH_3_), *ν*_*as*_(CH_2_), *ν*_*s*_(CH_3_) and *ν*_*s*_(CH_2_) stretches arising from lipid species within the cell, and located at 2955, 2920, 2870 and 2850 cm^−1^. Specifically, it appears that there is an overall increase in signal intensity for both the asymmetric and symmetric methylene *ν*(CH_2_) stretches with BaP treatment and a corresponding decrease in intensity for the methyl *ν*(CH_3_) asymmetric and symmetric stretches (in the second derivative spectra a peak minimum corresponds to the maxima intensity in the underivatised spectra). This trend is also observed in the synchrotron data as shown in Fig. 1b, which exhibits an overall increase in methylene intensity (shown as peak minima). The fingerprint region of the spectra in Fig. 1 is dominated, as expected, by the amide bands I (1639 cm^−1^) and II (1535 cm^−1^) arising from *ν*(C = O) stretching mode (Amide I) and δ(N-H) bending and *ν*(C-N) stretching modes of peptide linkages (Amide II), which typically represent total cellular protein. The fingerprint region also contains more subtle features, often appearing as small inflections of a peak more prominent in second derivative spectra, for example a change in DNA conformation can be determined by considering the position of the asymmetric phosphate stretching vibration observed at 1220 cm^−1^ in B-DNA and 1235–40 cm^−1^ in A-DNA, which is discussed later.

**Figure 4:**
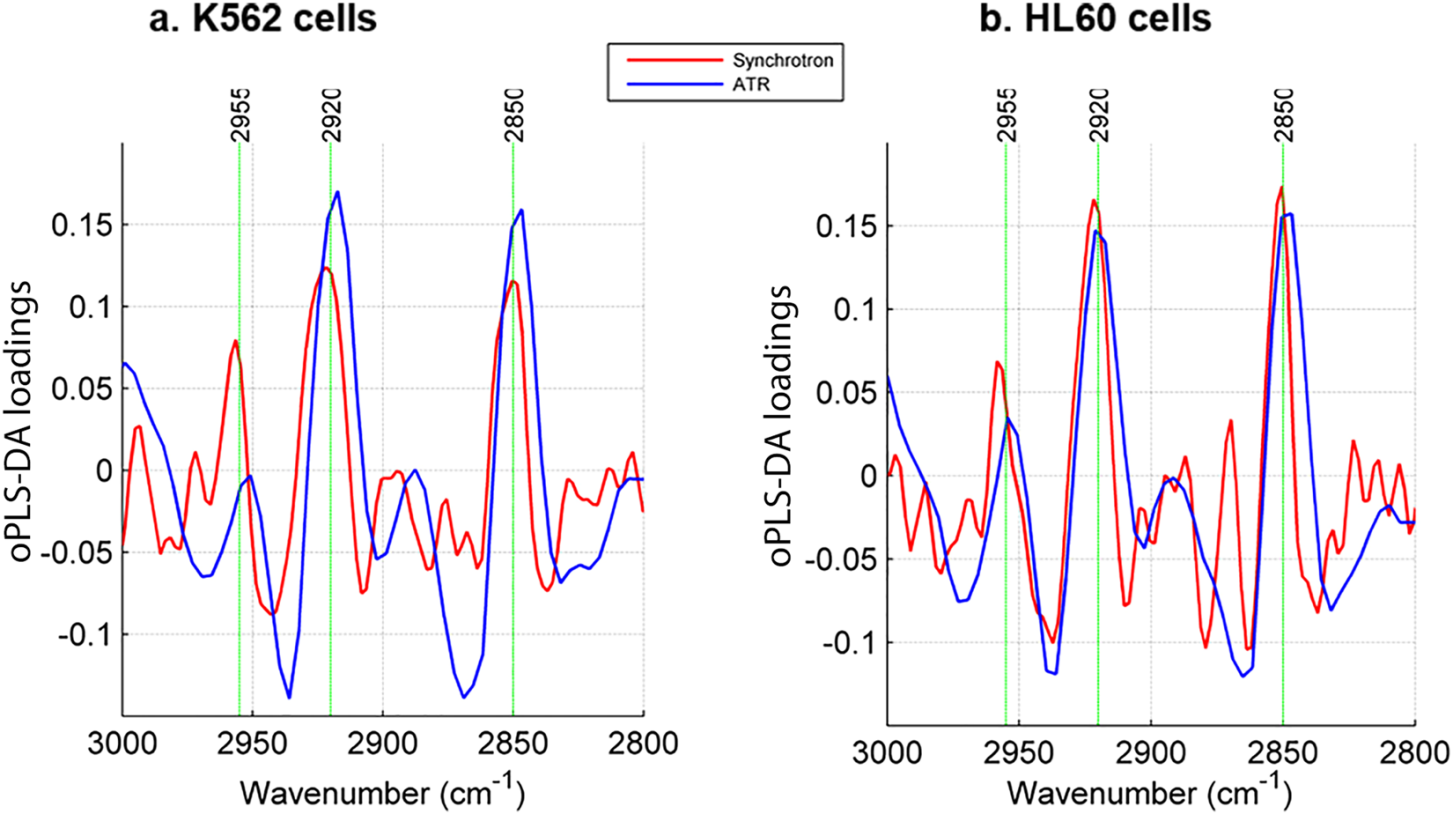
oPLS-DA loadings vectors overlaid for S-FTIR (red) and ATR-FTIR (blue) loadings in the high wavenumber lipid region, for K562 (**a**) and HL60 (**b**) cells.

An inspection of the first loadings vector from the oPLS-DA confirms that the main contribution to the separation observed in the scores plot is from the aforementioned methylene moieties, with prominent features observed in the high wavenumber region at 2920 cm^−1^ and 2850 cm^−1^. The first loadings vector from the oPLS-DA, with asymmetric and symmetric *ν*(C-H) stretches annotated can be seen in Fig. 4. Here, the loadings from both synchrotron and ATR spectra are overlaid showing consistent loadings and good correlation between data sets obtained with these two techniques. Loadings shown for the synchrotron spectra are noisier than spectra obtained with ATR likely due to differing amounts of biomaterial analysed; synchrotron data are averaged over a cell population of a few hundred cells for each cell line, however thousands of cells would have been present in the aliquot sampled and dried on the ATR element. Despite cells being hydrated for S-FTIR analysis and dehydrated in the case of ATR, both techniques produce concordant results and the predominant spectral changes arise from disruptions to cellular lipid composition. There is less of an observable contribution to the discrimination between control and drug treated cells in the lower wavenumber spectral region.

**Figure 5:**
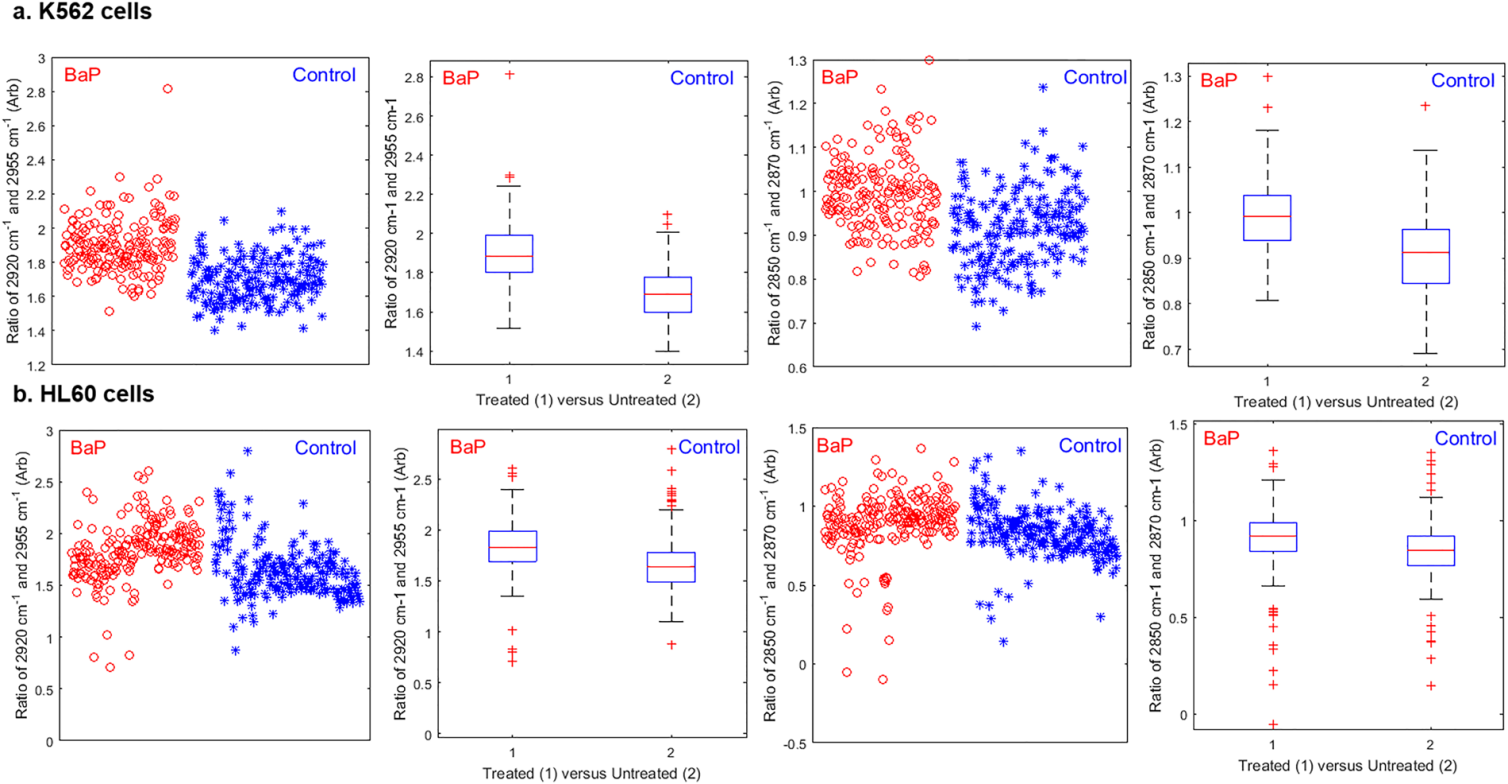
Box and whisker plots for significant changes (p < 0.05, see Supplementary Table S1) in peak area ratios of methylene to methyl stretching vibrations between control and 24 h BaP treated cells analysed by S-FTIR. a. shows peak area ratios 2920:2955 cm^−1^ for asymmetric C-H stretching and 2850:2870 cm^−1^ for symmetric C-H stretching for K562 cells and b. shows the same ratios for HL60 cells. The median of each box plot can be compared to describe the changes in lipid peak area ratios with drug treatment; with the maximum values, minimum values and outliers showing the spread in inter-cell variability within population sizes of 385 cells for K562 and 441 cells for HL60. Extreme outliers (n < 10) were removed from the plot for visual appearance, however all cells analysed were included in the data analysis.

The apparent change in methylene to methyl ratios in data acquired for BaP treated cells was further explored by extracting spectral peak areas for all single cells analysed with S-FTIR. Figure 5 shows peak area ratios for asymmetric and symmetric stretches of methylene and methyl moieties for K562 (Fig. 5a) and HL60 (Fig. 5b) cell populations. The box plots include all synchrotron spectra acquired, each point representing a cell; the spread therefore representing inter-cell variability across biological replicates spanning two trials. Data clearly show an increase in the methylene to methyl ratio with drug treatment for both asymmetric and symmetric stretches. A concurrent trend is also observed for ATR spectra acquired from cell pellets from 9 biological replicates, with data shown in Supplementary Figure S4.

PLS-DA confirms the grouping according to methylene to methyl peak area ratios for BaP treated and control cells and *p* values for each are detailed in Supplementary Table S1. The CH_2_:CH_3_ ratios proving to be significant in both cell lines (*p* = <0.05) for both synchrotron and ATR analyses.

A biological interpretation of these data is interesting since an increase in CH_2_:CH_3_ ratio indicates that lipids in drug treated AML cells show an overall change in saturation state with BaP treatment when compared with control cells. Lipid composition in drug treated cells may consist of a greater number of acyl chain double bonds at a chain end position or data could suggest increased lipid saturation in mid acyl chain positions. To gain further insight into this picture, Raman microspectroscopy was employed to image control and drug treated single cells and data for this is presented later.

Drawing conclusions on drug mode of action based on a consideration of just the lipid region of the spectra can, however, be somewhat short-sighted. Similar changes in lipid biochemistry can also be observed in cells undergoing various stages of cell death from necrosis through to apoptosis. The increase of lipids in this case is reportedly due to a number of cell membrane processes such as blebbing and vesicle formation, which can give rise to an increase in CH_2_ absorption observed in the spectra^45^. This implies that many drug-cell interaction studies, which report lipid changes, might in fact just be monitoring the death of a cell due to drug treatment and this has recently caused some concern in the spectroscopy community. In this study, the previously described BaP treatment is known to differentiate HL60 cells further and induce apoptosis in K562 cells, but not within the 24 hour treatment period used in this study^7^. When a cell enters apoptosis, the DNA shifts to a more disordered state, and the DNA signal in an infrared spectrum typically decreases^46^. A shift in the DNA band can also be observed in cells which have been treated with a drug known to intercalate with DNA, whose mode of action is such that base pairing is interrupted and unravelling from orderly B-DNA to the more disordered A-DNA form occurs^47^.

**Figure 6:**
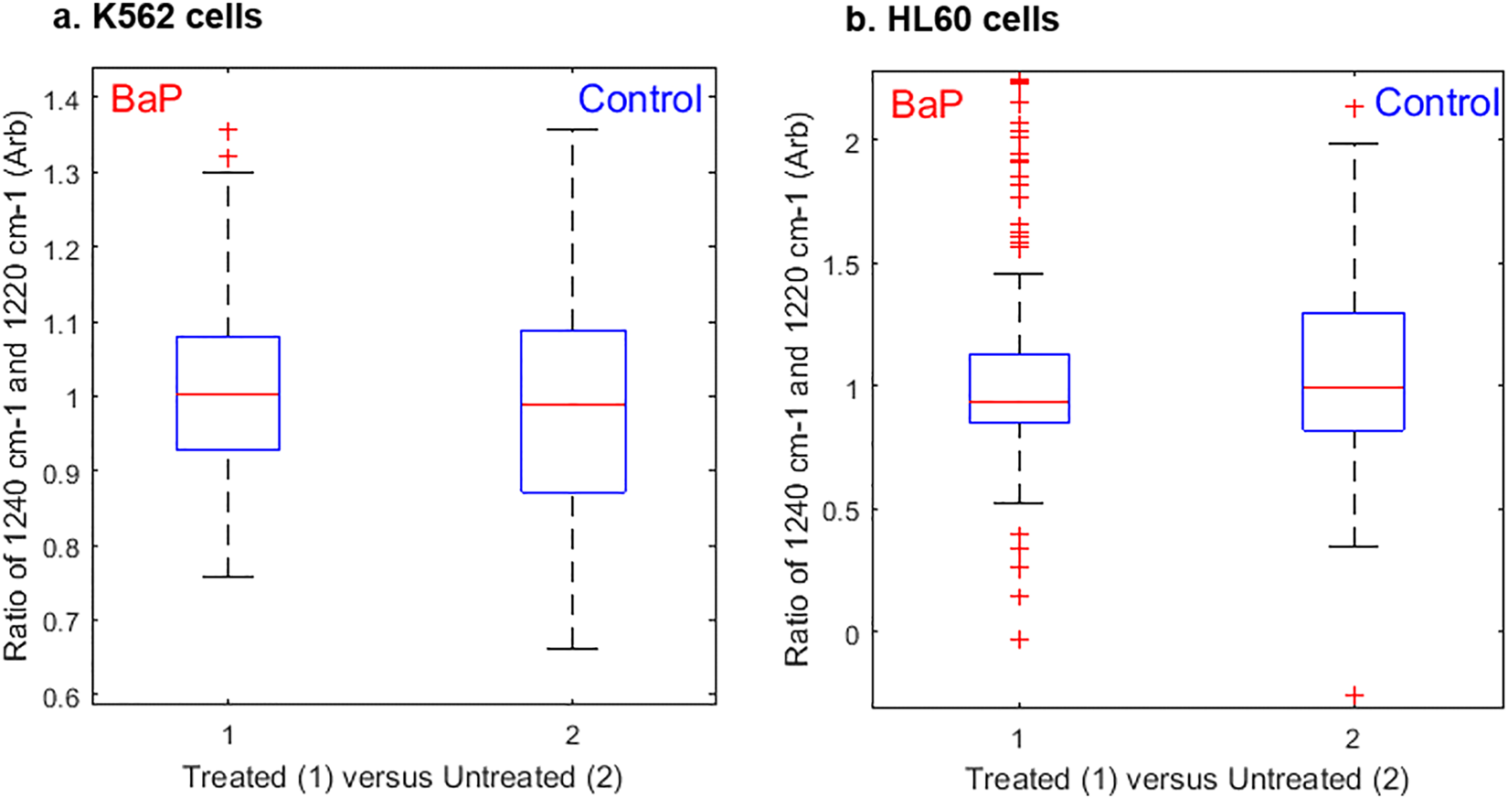
Box and whisker plots of peak height ratios for the asymmetric phosphate stretch at 1240:1220 cm^−1^ calculated for the entire cell population of K562 cells (**a**) and HL60 cells (**b**) analysed by S-FTIR. The median line indicates no significant shift in asymmetric phosphate stretch peak position between control and drug treated cells for either cell class.

 Care must be taken when interpreting the data to ascertain if the spectra show evidence of drug induced biochemical changes or are indicative of cell death. Here, the major DNA peaks have been explored to assess if one of the targets of BaP could in fact be DNA and to confirm that what was being monitored in this study was not simply cell death. One of the vibrations as a marker for the latter hypothesis is the carbonyl vibration for protein linking in the amide I band at 1657 cm^−1^ which has been reported to significantly decrease with apoptosis^48^. A visual inspection of the spectra in Fig. 1 indicates no obvious change in amide I signal intensity with BaP treatment. Perhaps more diagnostic though is the position of the asymmetric phosphate stretch of DNA which is reported to shift from 1220 cm^−1^ to 1240 cm^−1^ with a conformation change from B-DNA to A-DNA^30^, a phenomenon which can occur during drug treatment and also during cell dehydration. Fully hydrated DNA exists in the B-conformation and one of the main markers for a B- to A-DNA transition is the shift of *ν*_asym_(PO_2_^−^) to a higher wavenumber. As such, this change can only be confidently observed in synchrotron spectra for which cells are hydrated during data acquisition. Figure 6 shows peak area ratios between control and drug treated cells for the asymmetric phosphate stretch of DNA, to look for a shift in peak maxima indicating a change in DNA conformation. For this scenario we have a null hypothesis, with DNA signals not responding in a manner indicative of cell death. The absence of a significant change in this peak area ratio is further confirmed in Supplementary Table S1, with the 1240:1220 peak ratios showing very high *p* values, thereby not being significant. This information, coupled with the apparent absence of change for the amide I peak intensity strongly suggests that DNA is not a target of BaP therapy. This is of key significance from an *in vivo* biological perspective, because it could explain why the drug combination BaP showed little or no haematological toxicity to healthy cells in a small cohort of patients recruited for clinical trial^4^. If BaP was interfering with the DNA of a cell, one would expect that to be a fairly non selective process and thus signs of DNA disorder across all cells might be expected.

### Raman Mapping of single cells to further characterise BaP-induced lipid changes

**Figure 7:**
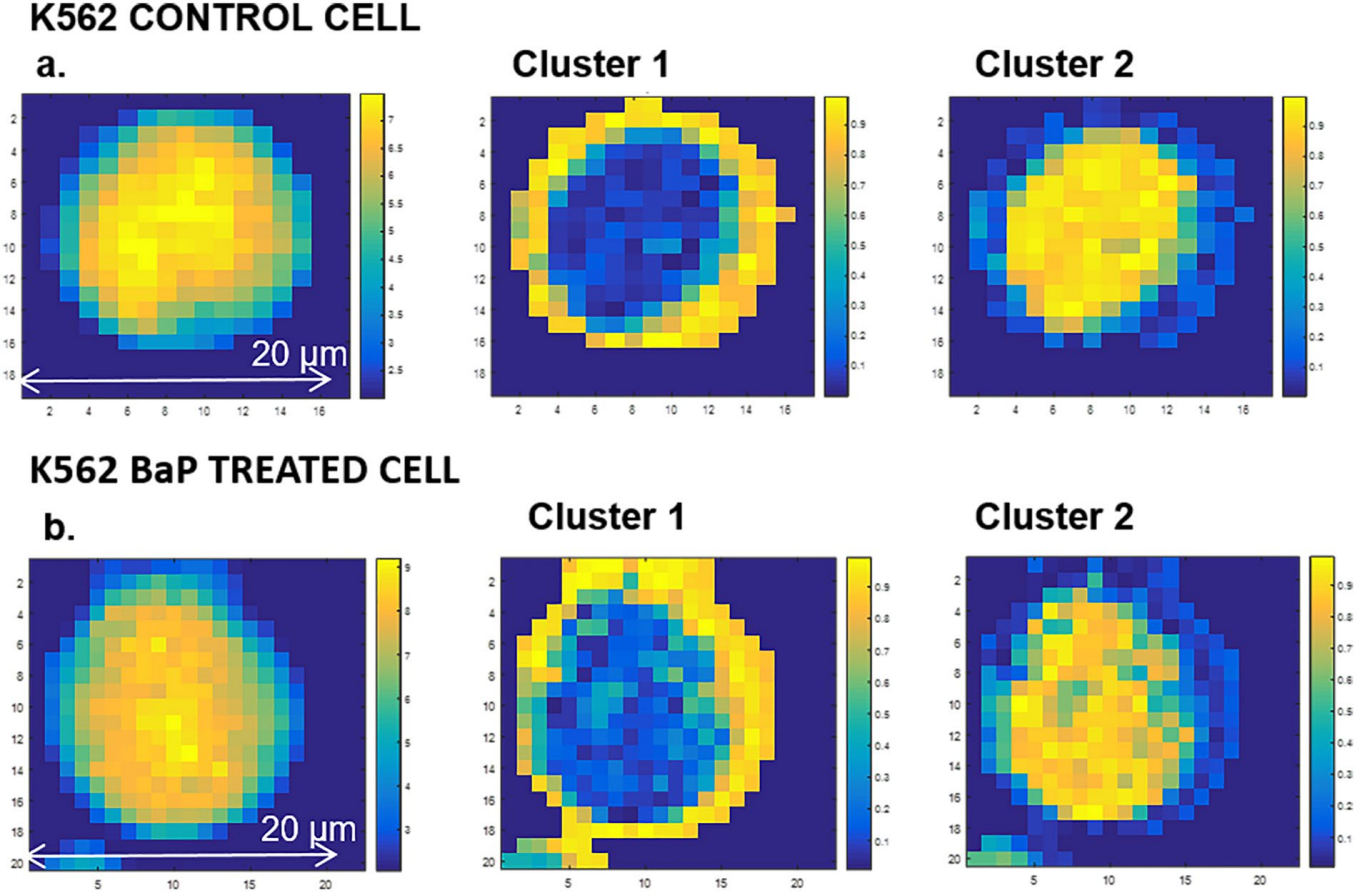
FCM images of a representative control and BaP treated K562 formalin fixed cell.

Raman mapping was employed to further probe drug-induced biochemical changes observed with FTIR and to provide sub-cellular spatial information regarding these changes. The resulting data matrix for each cell map obtained was refined by removing background pixels by evaluating the signal-to-noise ratio of the Raman spectra. A cut-off threshold of 2 efficiently removed all the background pixels as shown in Fig. 7. Single cell images of cell features were derived using fuzzy c-means clustering (FCM) analysis on all spectra obtained from K562 formalin fixed control and drug treated cells (*n* = 6 and *n* = 5 respectively). In this process, each pixel is associated with a fuzzy membership value so that the gradient from one cell compartment to another can be well presented. Figure 7 shows a representative K562 control cell (Fig. 7a) and drug treated cell (Fig. 7b) with the left most images showing the single cell image refined through the removal of background pixels by evaluating the signal-to-noise ratio of the Raman spectra. The cell membrane is clearly defined (yellow) around the outer perimeter of the cell in Cluster 1 and internal cell structures clearly observable (yellow) in Cluster 2.

**Figure 8:**
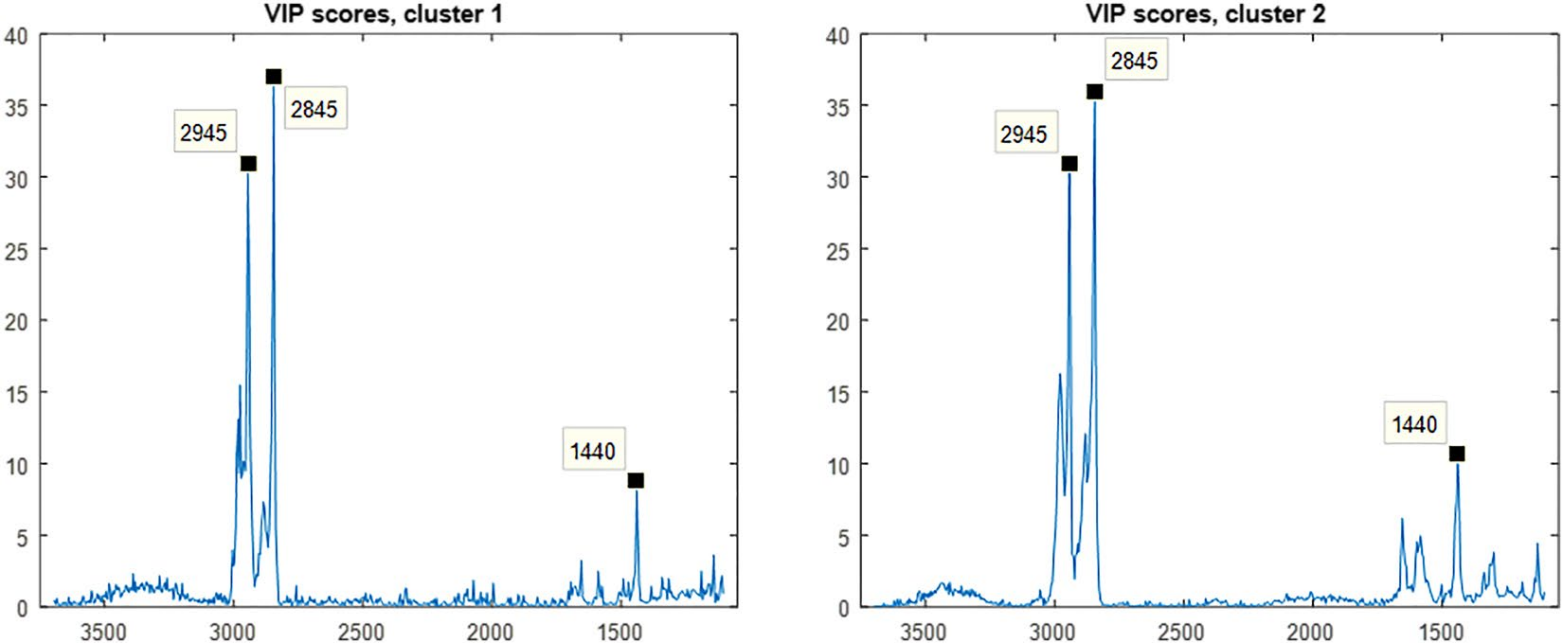
Variable Importance in Projection (VIP) scores for PLS-DA of K562 control and drug treated cells showing three Raman lipid peaks.

The confusion matrices for the PLS-DA computed across the entire spectral region for all 11 K562 formalin fixed cells are detailed in Supplementary Table S2, with correct classification rates of 95.23% for BaP treated cells and 89.11% for control cells of combined clusters (all cell spectra). Despite there being no obvious changes by eye when comparing the images with each other, the VIP PLS-DA scores plots shown in Fig. 8 clearly highlight three distinct Raman peaks as key to this classification which all arise from cellular lipid changes with BaP treatment. Mapping the spatial distribution of spectral features across single cells has identified that the aforementioned lipid changes appear to be consistent across the whole cell, with no obvious regional differentiation between membrane lipids and intra-cellular lipids.

### Biological interpretation of observed cellular lipid changes

All three spectroscopic techniques employed in this study revealed an increase in the CH_2_:CH_3_ ratio of lipid peaks in the high wavenumber spectral region of drug treated cells which indicates a change in cellular lipid saturation state with BaP treatment and demonstrates good consistency between the analytical techniques. From the FTIR data alone it is not possible to infer specifically what lipid saturation change this refers to. However, in the case of the Raman spectra the peak observed at 1440 cm^−1^ decreases (reportedly proportional to total lipid saturation) indicating an increase in lipid unsaturation with BaP treatment. This band would also have contributions from methyl and methylene modes from amino acid side chains of proteins but the fact that there is also a correlation with the increase in intensity of the bands in the methyl/methylene stretching region (3000–2800 cm^−1^) indicates that the major changes are the result of changes in lipids and not proteins. This is consistent with our previous mass spectrometry lipidomics study where BaP treated AML cells had more unsaturated phosphatidylcholine than control cells^8^. An explanation for this altered lipid saturation is that BaP has been shown to decrease *de novo* fatty acid synthesis—synthesis of saturated and monounsaturated fatty acids and increase uptake of polyunsaturated fatty acids from the media^8^.

One novel aspect of these data is the visualisation of increased lipid unsaturation at the single cell level and data suggest that the BaP-induced change in saturation state is uniform across the cell. Increasing lipid unsaturation typically renders these biomolecules more fluid which has implications for cancer cell biology and survival. The phenomenon of high rates of *de novo* lipogenesis observed in cancer cells enables them to synthesise new cell membranes during rapid proliferation and it also allows cancer cells to be more independent in terms of their energy supply and biochemical need for growth. Enhanced lipogenic phenotype is characterised by lipids with saturated or mono-unsaturated acyl chains in a number of different cancer cell lines which is significant in terms of protecting them from lipid peroxidation and apoptosis^49^. During lipid peroxidation, free radicals remove electrons from cell membrane lipids, producing oxidised lipid species and small molecule reactive oxygen species (ROS), which could jeopardise the stability and longevity of a tumour cell^49^. This is pertinent because BaP induces high levels of ROS, which is associated with its anticancer effect^7^. Therefore the actions of BaP may involve both an increase in production of ROS and an increase in cell susceptibility to ROS. Membrane rigidity (arising from increased lipid saturation) is also implicated in cell resistance to anti-cancer drugs by impairing uptake of drugs through the membrane^50^ and any increasing lipid unsaturation observed within cells could be a mechanism to preventing drug resistance.

Considering the important role that saturated lipids play in cancer cell function and survival, spectroscopic data reported here suggests that the BaP-induced increase in lipid unsaturation observed is likely to play a role in its anti-cancer activity.

## Conclusion

Employing a multi-platform spectroscopic approach to probe the action of BaP on HL60 and K562 cell lines affords a comprehensive picture of the effect of drug therapy on the biochemical nature of the cells and allows us to further understand the targets of BaP. Data reported for S-FTIR, ATR-FTIR and Raman microspectroscopy strongly indicate that lipid biochemistry is a significant target of BaP; furthermore, the ability of S-FTIR to probe cells in a hydrated state enabled ‘drug-DNA’ interaction to be probed and indicated that DNA is unlikely to be a target of this therapy.

A significant increase in methylene functionality with drug treatment indicates that AML cells with increased unsaturation post-BaP treatment are representative of the more typical biochemistry of non-cancerous cells. Raman microspectroscopy supported the findings of FTIR and added a further dimension to the study by providing spatial information of lipid distribution which suggested that BaP-induced saturation change is uniform across a single cell.

## Materials and Methods

### Cell Culture

Acute myeloid leukaemia (HL60 and K562) cell lines were cultured in RPMI 1640 medium (+L-Glutamine) supplemented with 10% (v/v) fetal bovine serum (FBS), 1% (v/v) penicillin-streptomycin (Life Technologies, ThermoFisher) and maintained in exponential growth (between 0.25–1.5 × 10^6^ cells/mL) at 37 °C with 5% CO_2_. Cells were cultured *in situ* at the Australian Synchrotron (Clayton, Victoria) for one week prior to experimentation.

### Drug Treatment

Cells were seeded at 0.5 × 10^6^ cells/mL in T-25 flasks (vented) and treated with 0.5 mM bezafibrate in DMSO and 5 µM medroxyprogesterone acetate in ethanol (combination treatment denoted ‘BaP’)^7^. Control flasks (from the same seeding flask as drug treated cells) received equal volumes of ethanol and DMSO. Cells were incubated for 24 hours at 37 °C with 5% CO_2._

### Preparation of Cells

Control and BaP treated cell suspensions of HL60 and K562 in growth media were removed from the incubator at 24 hours and centrifuged at 500 × *g* for 5 min. The supernatant was discarded, leaving a small volume of liquid in the cell pellet.

*For Synchrotron-FTIR analysis*, minimal sample preparation was required; the solution was agitated gently to release the cell pellet with a pipette tip and 2 µL of the concentrated cell suspension was removed and gently placed on a CaF_2_ disc seated in a purpose-built microfluidics sample accessory device as previously described^51^. This ensured cells were in a fully hydrated state for analysis while the microfabricated gasket thickness was selected to ensure the cells were gently held between the upper and lower windows of the chamber and remained stationary during measurement.

*For ATR-FTIR analysis*, the cell pellet was washed twice with physiological saline solution with centrifugation performed between washes. The remaining cell pellet was treated as stated above with 2 µL of the concentrated cell suspension being placed directly onto the diamond ATR window and allowed to air dry thoroughly before recording spectra.

*For Raman analysis*, the cell pellet was re-suspended in Dulbecco’s phosphate buffered saline (PBS) solution, 500 µL (Sigma-Aldrich). The solution was well agitated, transferred into a microcentrifuge tube and centrifuged at 500 × *g* for 5 min using a small bench-top centrifuge. The supernatant was removed and the cell pellet was washed 3 times with PBS, 500 µL. The cell pellet was resuspended in approximately 50 µL (containing approximately 3 million cells) and spotted onto a CaF_2_ disc and the excess suspension drawn off the disc to leave a sparse monolayer of cells remaining. Once the surface of the disc had dried, excess formalin (10% formalin solution, buffered pH 7.0, VWR) was spotted onto the surface to fix the cells. The formalin was left on the disc surface for 15 min then removed by gently shaking off the excess and the fixed cells were subsequently washed with 3 × PBS, 500 µL and air dried prior to analysis.

### Synchrotron-FTIR Measurements

Synchrotron-FTIR measurements were recorded using the FTIR microspectroscopy beamline at the Australian Synchrotron. For the microspectroscopy beamline the beam is directed to a Bruker Hyperion 3000 IR microscope (Bruker Optics GmbH., Ettlingen, Germany) equipped with a liquid-nitrogen-cooled mercury-cadmium-telluride (MCT) detector with a × 36 IR objective (NA = 0.5). The Hyperion 3000 microscope is coupled to a Bruker Vertex 80 v spectrometer and data collection was carried out using Bruker’s *OPUS version 6.5* software, with an additional 3D analysis package (Bruker Optics GmbH., Ettlingen, Germany). The Hyperion microscope and the sample were purged with dry nitrogen gas to minimize water vapour contributions and carbon dioxide in the spectra. Spectra were recorded in transmission mode in the region 4000–900 cm^−1^ with a spectral resolution of 6 cm^−1^ using an aperture size of 10 × 10 µm. This aperture size was chosen to reflect the typical cell sizes of approx. 10–14 µm (HL60) and 12–16 µm (K562). Background spectra of a cell free region containing only cell culture media were taken every 5 spectra to account for any non-cell specific contributions to the signal and beam current fluctuations and 64 interferograms were co-added per measurement in order to obtain a good signal-to-noise ratio. Spectra were collected from at least 385 single cells for each cell line, which accounts for biological replicates and analysis on different days. The exact number of cells studied for each cell line is specified in the legend of Fig. 1.

### ATR-FTIR Measurements

ATR-FTIR analyses were performed on an Agilent 4500a portable FTIR spectrometer equipped with a triple reflection diamond ATR sample interface. Spectra were collected at 4 cm^−1^ resolution with 64 co-added interferograms corrected against a clean diamond background. For each cell line, 9 biological replicates were analysed for both drug treated and control cells.

### Raman Measurements

Raman spectroscopic analysis was performed using a Renishaw inVia Raman microscope (Renishaw plc., Wooton-under-edge, UK). Raman spectra and maps were obtained; using a 532 nm excitation beam, ~30 mW at the sample, ×50 objective and exposure times of 10 s for point spectra and 6 accumulations of 1 s (6 s in total) for mapping. StreamLineHR mapping (which uses a laser-spot focus) was carried out in high confocal mode, at a step size of 0.5 μm. Data were processed using algorithms in the Renishaw WiRE software and using scripts written in MATLAB (The Mathworks Inc. Natik, U.S.A) during which the spectral hypercube of each map was analysed to produce images.

### FTIR Data Analysis

Pre-processing of the spectra was carried out in MATLAB. For both cell lines, all spectra acquired were used in the data modelling. Raw spectra were corrected for baseline fluctuations and any scaling effects due to different pathlengths, using extended multiplicative signal correction (EMSC)^52^. To avoid introducing any further artefacts into the data set, no further pre-processing algorithms were employed. Following EMSC correction, the spectra were cropped to the specific regions of interest to be analysed (3100–2800 cm^−1^ for the lipid region and 1800–1100 cm^−1^ for the fingerprint region). ATR-FTIR spectra were processed as the second derivate to enhance spectral features. All spectral assignments have been made according to widely cited literature references^19, 53, 54^.

### Statistical Analysis

#### Orthogonal Partial least squares-discriminant analysis (oPLS-DA)

Prior to the oPLS-DA, spectra data matrix was pre-processed using a) Savitzky-Golay filtering based second derivative (order: 2, window: 9 points), b) standard normal variate spectra normalization, c) Pareto scaling variable pre-processing and d) mean centring. Data processing was performed employing MATLAB using the routines available on the *PLSToolbox* package from *Eigenvector Research Inc*. (Manson, USA).

#### Bootstrapping Validation

In order to validate the models generated by partial least squares-discriminant analysis (PLS-DA) bootstrapping with replacement was used for the synchrotron FTIR spectra. In this resampling process a 1000 bootstraps were undertaken and in each bootstrap each training set contained on average 63.2% of all samples, and each test sets included the remaining 36.8% samples. Statistics were then generated for the 1000 models in terms of correct classification rates and confusion matrices. This technique gives well represented estimates of the average model and is therefore less likely to be biased^55^.

### Raman Data Analysis

Raman maps for 5 control cells and 6 drug treated cells were pre-processed in WiRE software (Renishaw plc.) for cosmic ray removal and further manipulated according to in-house scripts in MATLAB. Signal-to-noise (SNR) assessment was performed using Morphological Scores (MoS)^56^. Fuzzy c-means clustering algorithm was employed to image features of the cell and subsequently PLS-DA was able to differentiate between the two cell classes. The PLS-DA models were validated by using a *k*-fold cross double cross-validation (CV)^57^ where *k* is the number of cells, i.e. the spectra of an individual cell were held out as the test set and the model was built on the remaining spectra. The model was then applied to the test set spectra to predict their class membership. This procedure was repeated *k* times until each cell had been held out as test set for once. Within each CV procedure, the number of PLS-components was chosen by employing another *k*-fold CV on the training set and the one generating the best results on the internal validation set was chosen. A summary of Raman image data analysis can be seen in Supplementary Figure S5.

## Electronic supplementary material

Supplementary Information
