# Supplementary material for: Probing the action of a novel anti-leukaemic drug therapy at the single cell level using modern vibrational spectroscopy techniques
Source: Sci Rep. 2017 Jun 1;7:2649. doi: 10.1038/s41598-017-02069-5 (PMC5453947; doi:10.1038/s41598-017-02069-5)
Supplement: Supplementary file 1 — Supplementary Information [file 41598_2017_2069_MOESM1_ESM.pdf]

Probing the action of a novel anti-leukaemic drug therapy at the single cell level using modern vibrational spectroscopy techniques

Joanna L. Denbigh[a,b], David Perez-Guaita[c], Robbin R. Vernooij[c], Mark J. Tobin[d], Keith R. Bambery[d], Yun Xu[a], Andrew D. Southam[e], Farhat L. Khanim[e], Mark T. Drayson[f], Nicholas P. Lockyer[a], Royston Goodacre[a] and Bayden R. Wood[c*]

Supplementary Information

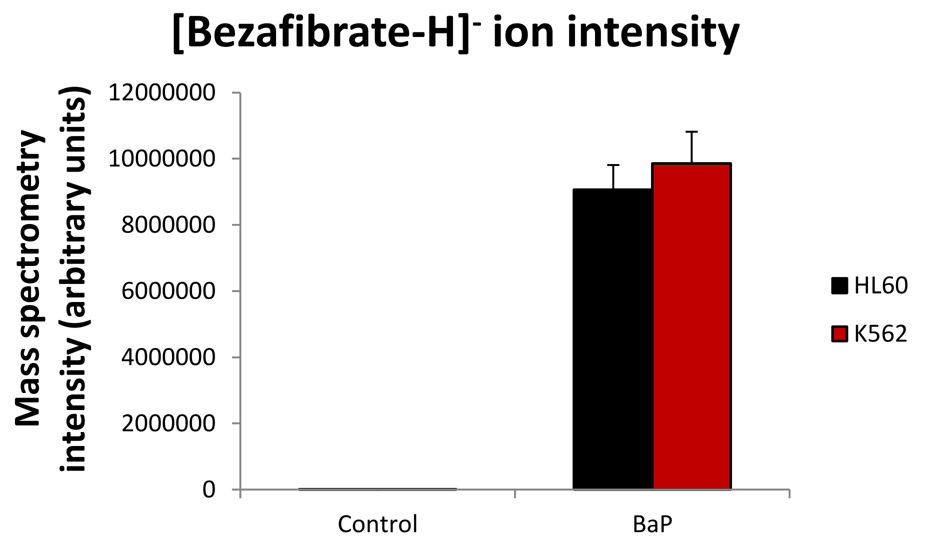

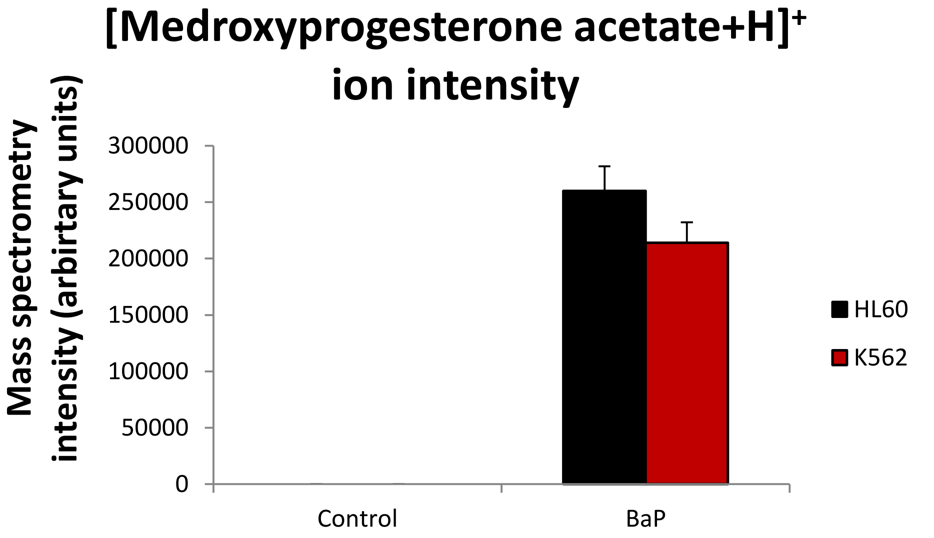

Supplementary Figure S1: **The mass spectral intensity of bezafibrate and medroxyprogesterone acetate ions in the metabolite extracts of drug treated (BaP) and control HL60 and K562 cells (n≥4). Cells were drug treated for 24 h (see methods section in the main manuscript). Metabolite extraction and FT-ICR direct infusion mass**

spectrometry was carried out as in Southam *et al.* 2015[1]. Bezafibrate was identified as the $[M-H]^-$ ion (negative ion mode, accurate *m/z* = 360.10081) and medroxyprogesterone acetate was identified as the $[M+H]^+$ ion (positive ion mode, accurate *m/z* = 387.25298). In all cases the mass error of the measured peak in the cellular extract was within ±1 ppm of the accurate mass.

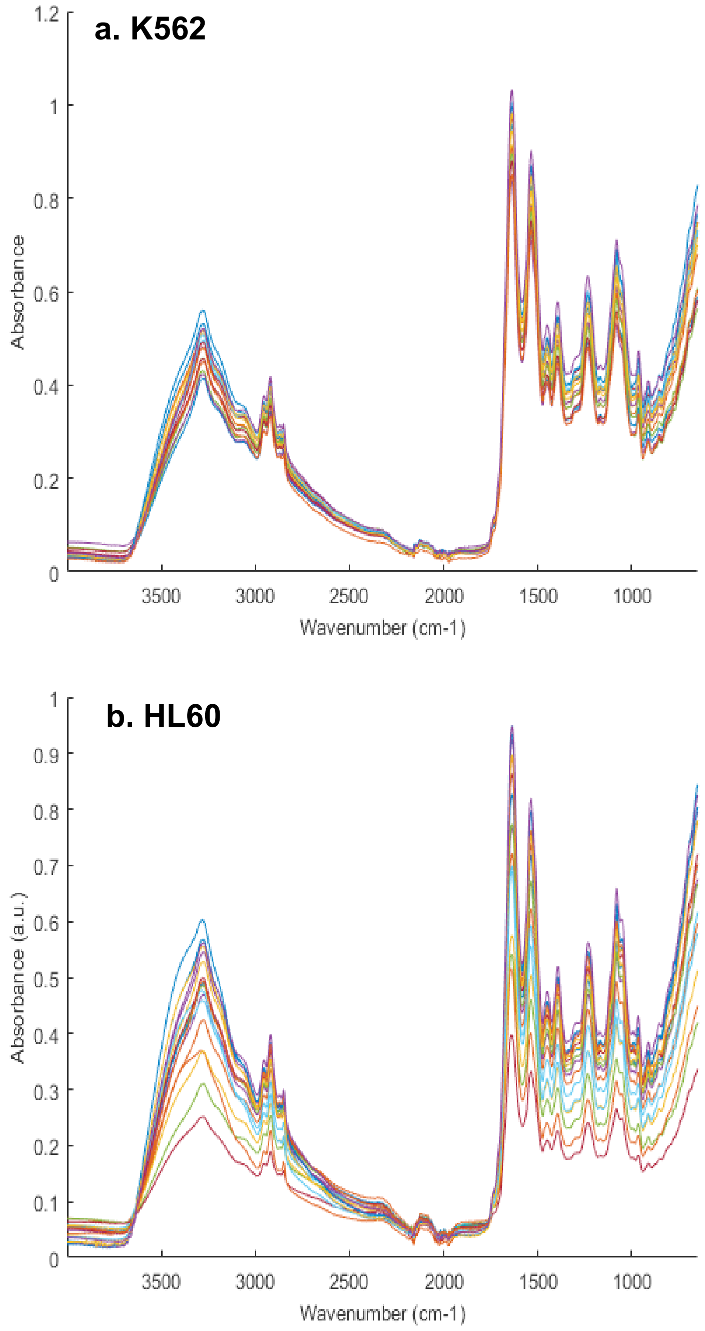

Supplementary Figure S2. Raw ATR spectra for cell pellets of K562 cells (a) and HL60 cells (b).

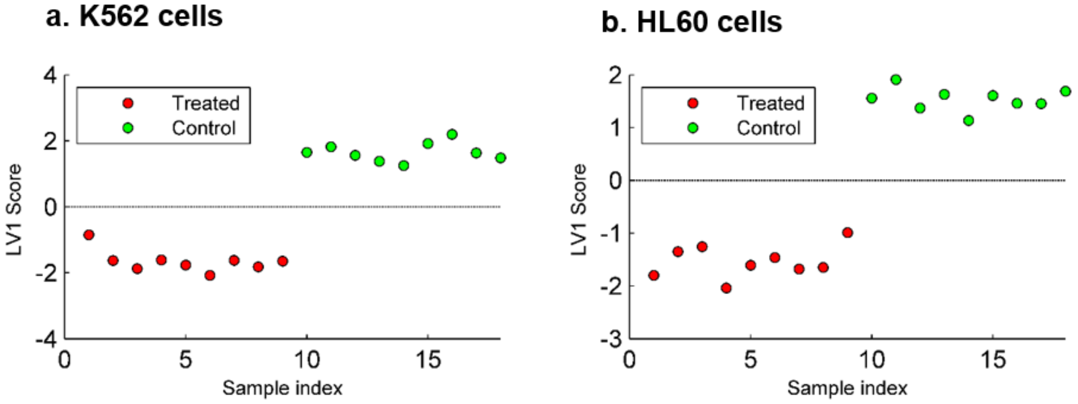

Supplementary Figure S3. Scores plots for the first oPLS-DA component for ATR spectra of control (green) and BaP treated (red) cells. Each point represents a replicate of cell pellet of K562 cells (a) and HL60 cells (b).

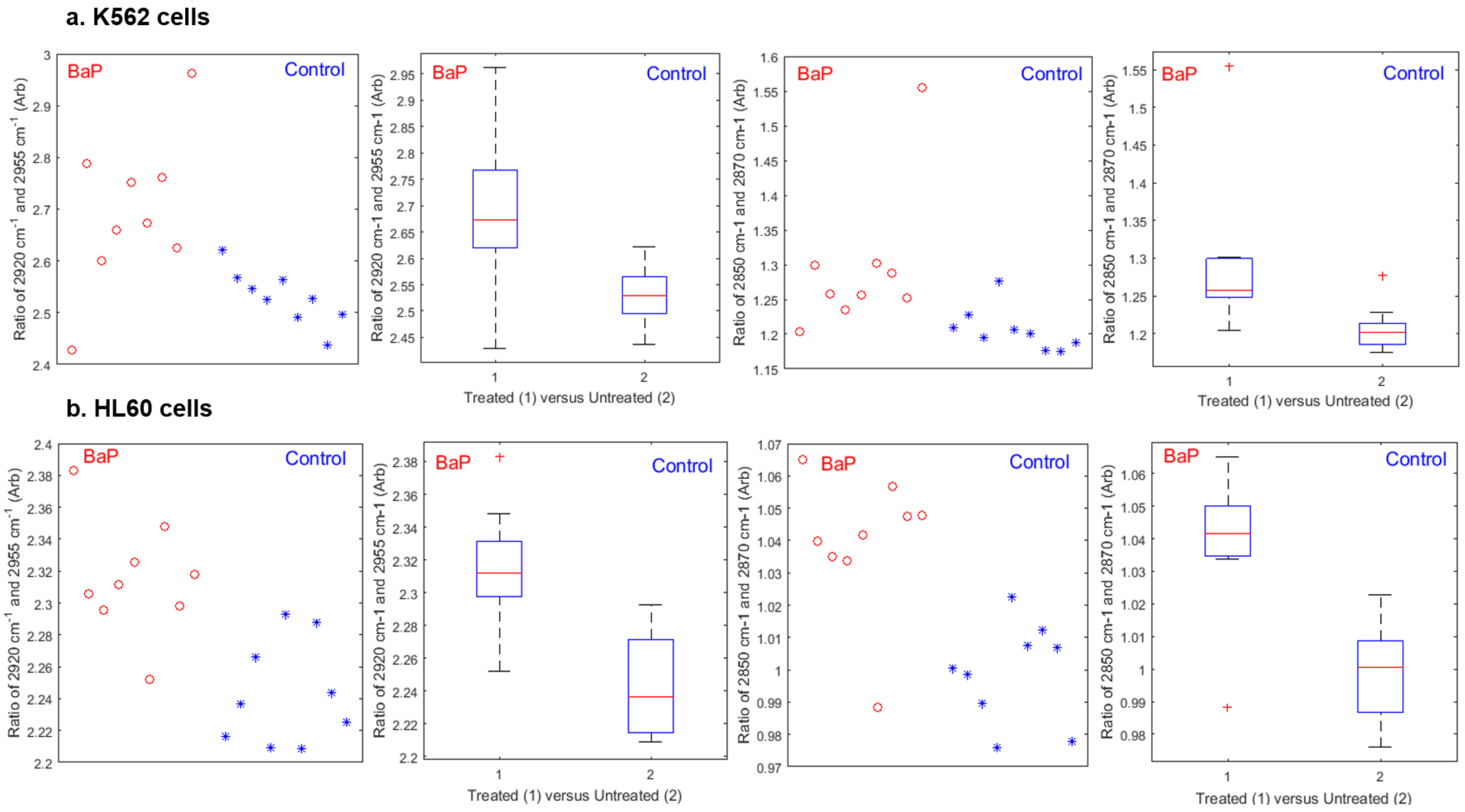

Supplementary Figure S4. **Box and whisker plots for significant changes (*p* <0.05, see Supplementary Table S1) in peak area ratios of methylene to methyl stretching vibrations between control and 24 h drug treated cell pellets analysed by ATR. a. shows peak area ratios 2920:2955 cm^{-1} for asymmetric C-H stretching and 2850:2870 cm^{-1} for symmetric C-H stretching for K562 cells and b. shows the same ratios for HL60 cells. The trend observed is concurrent with synchrotron FTIR single cell data shown in Figure 5.**

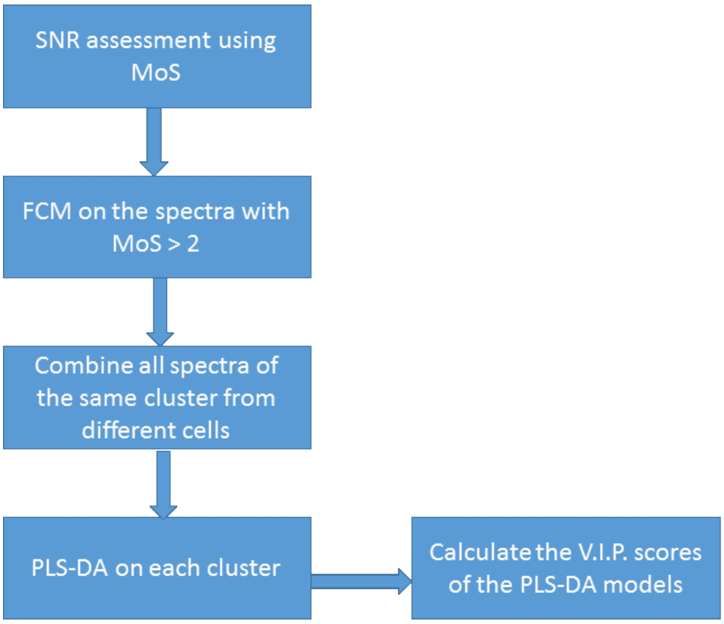

Supplementary Figure S5. Raman map data analysis work flow.

S-FTIR (raw, heights)	K562	HL60
CH_2/CH_3 from 2920 cm^{-1} : 2955 cm^{-1}	1.0757×10^{-20}	6.2607×10^{-5}
CH_2/CH_3 from 2850 cm^{-1} : 2870 cm^{-1}	2.4078×10^{-13}	2.1×10^{-3}
1240 cm^{-1} : 1220 cm^{-1} ratio	0.6660	0.7681
**ATR-FTIR (2nd deriv, heights)**	**K562**	**HL60**
CH_2/CH_3 from 2920 cm^{-1} : 2955 cm^{-1}	0.0259	0.0148
CH_2/CH_3 from 2850 cm^{-1} : 2870 cm^{-1}	0.0539	0.0331
1240 cm^{-1} : 1220 cm^{-1} ratio	0.1981	0.7738

Supplementary Table S1. Student t-test *p* values to quantify the group separation in the PLS-DA scores of each cell line analysed by S-FTIR and ATR-FTIR.

Cluster 1	BaP	CON
BaP	88.38%	11.62%
CON	23.66%	76.34%
**Cluster 2**	**BaP**	**CON**
BaP	98.38%	1.62%
CON	7.46%	92.54%
**Combined**	**BaP**	**CON**
BaP	95.23%	4.77%
CON	10.89%	89.11%

Supplementary Table S2. PLS-DA K-fold-cross-validation model on 11 K562 cells. Rows represent predicted classification and columns represent experimental.

References

1 Southam, A. D. *et al.* Drug Redeployment to Kill Leukemia and Lymphoma Cells by Disrupting SCD1-Mediated Synthesis of Monounsaturated Fatty Acids. *Cancer Res.* **75**, 2530-2540, doi:10.1158/0008-5472.can-15-0202 (2015).
